# Supplementary figures and images for: Dopaminergic Modulation of Orofacial Mechanical Hypersensitivity Induced by Infraorbital Nerve Injury
Source: Int J Mol Sci. 2020 Mar 12;21(6):1945. doi: 10.3390/ijms21061945 (PMC7139594; doi:10.3390/ijms21061945)

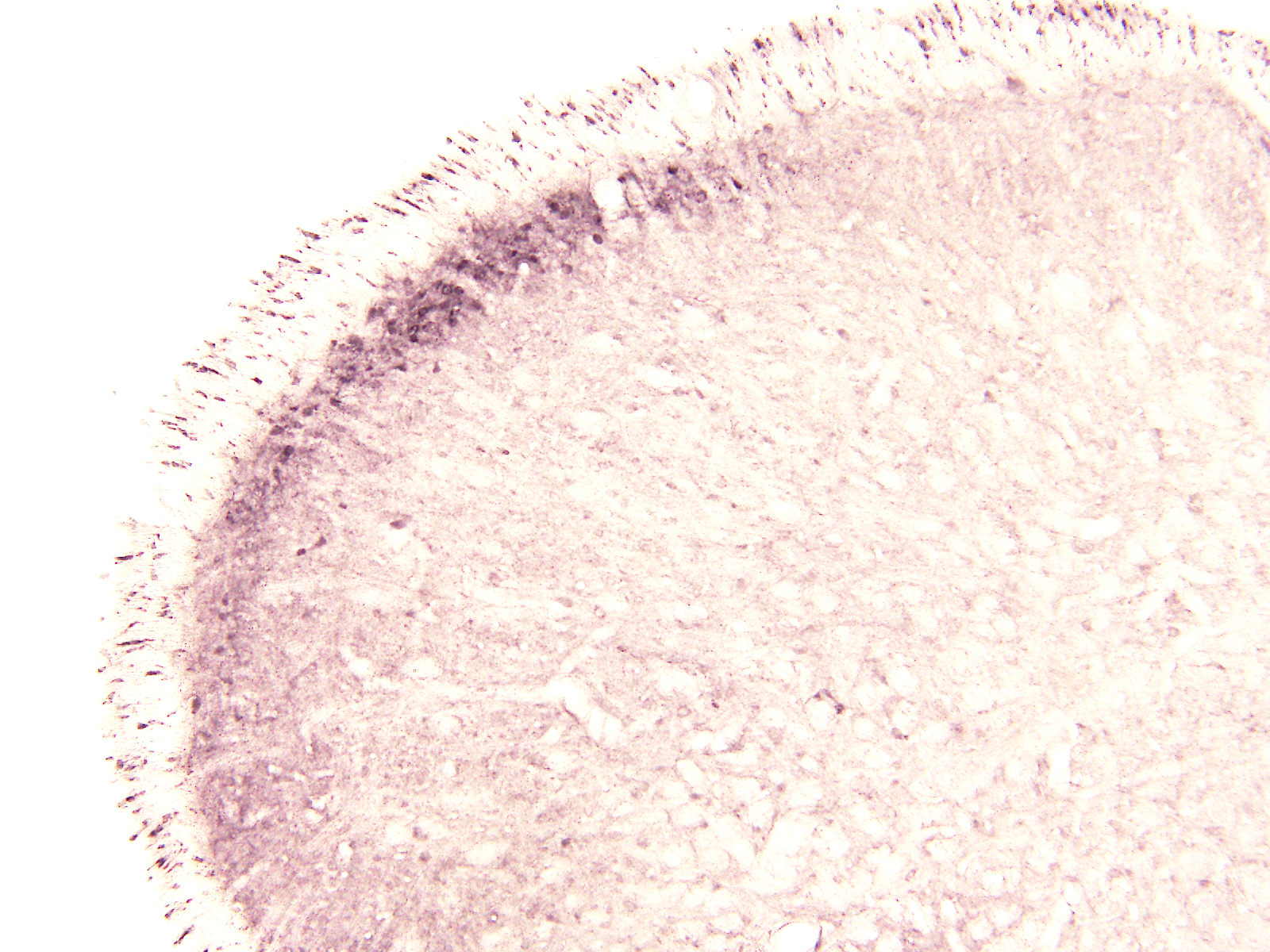

Supplement: Supplementary file 1 [file ijms-21-01945-s001.zip › Supplementary files/Figure S1.TIF]

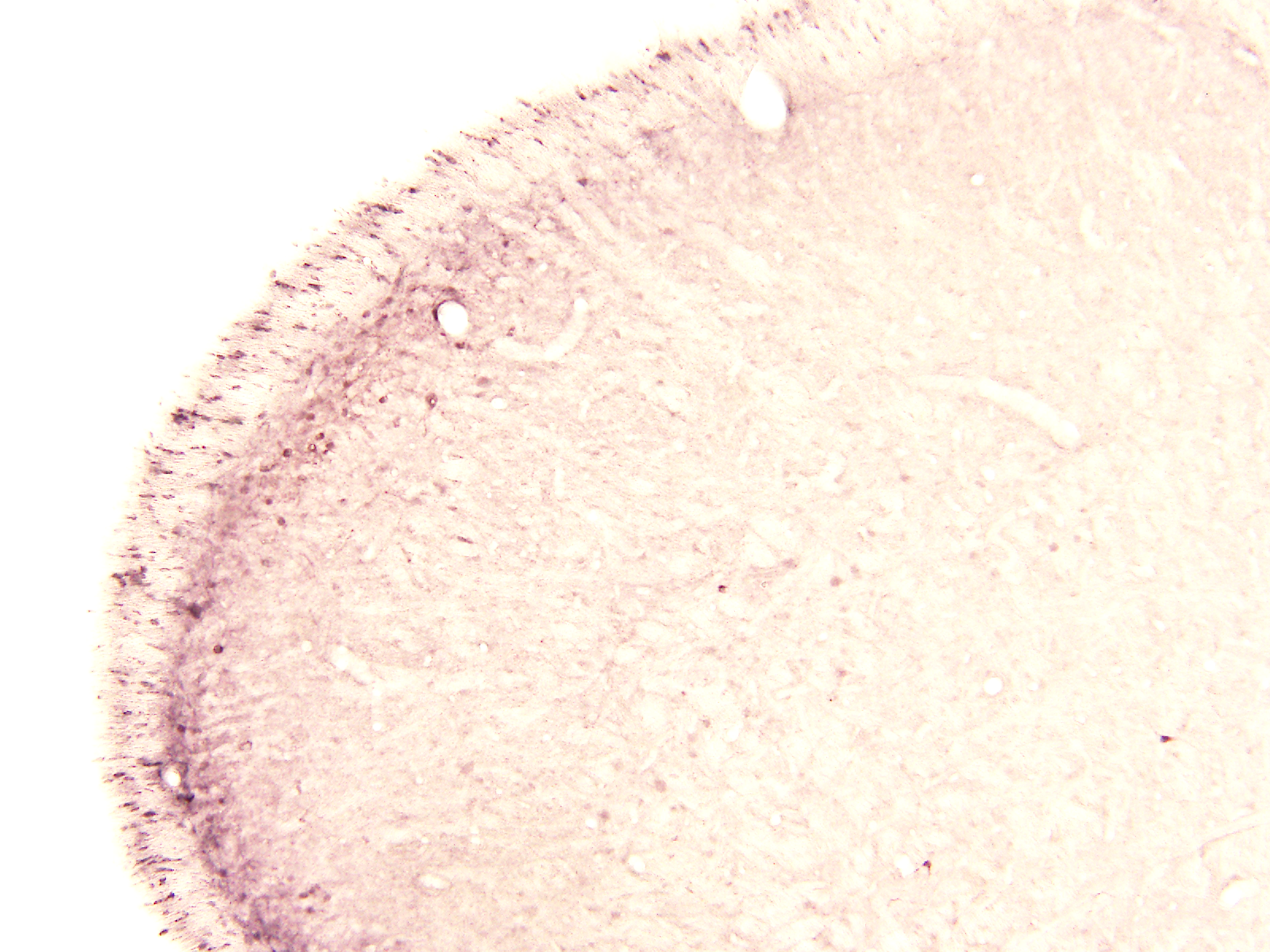

Supplement: Supplementary file 1 [file ijms-21-01945-s001.zip › Supplementary files/Figure S10.TIF]

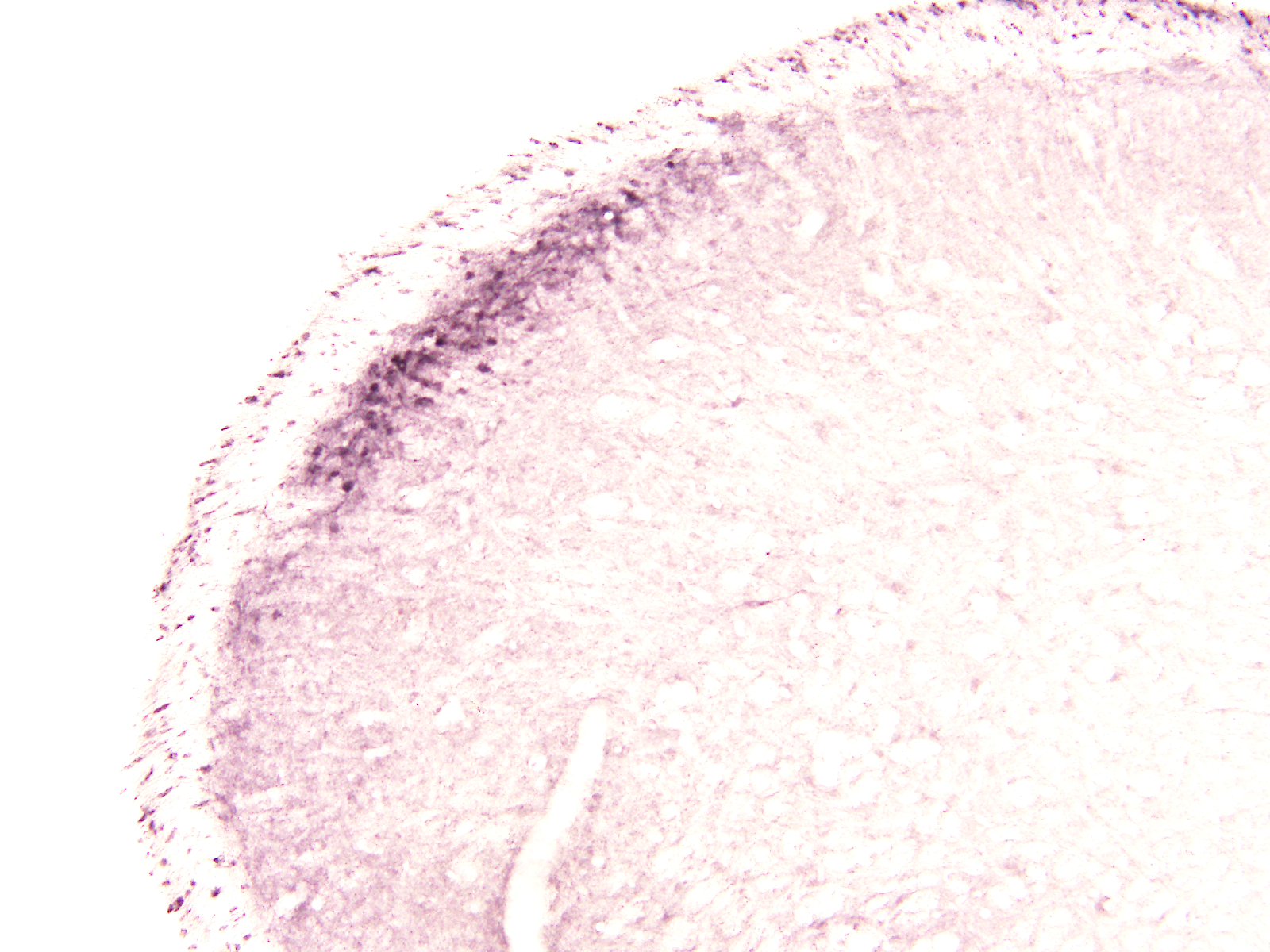

Supplement: Supplementary file 1 [file ijms-21-01945-s001.zip › Supplementary files/Figure S11.TIF]

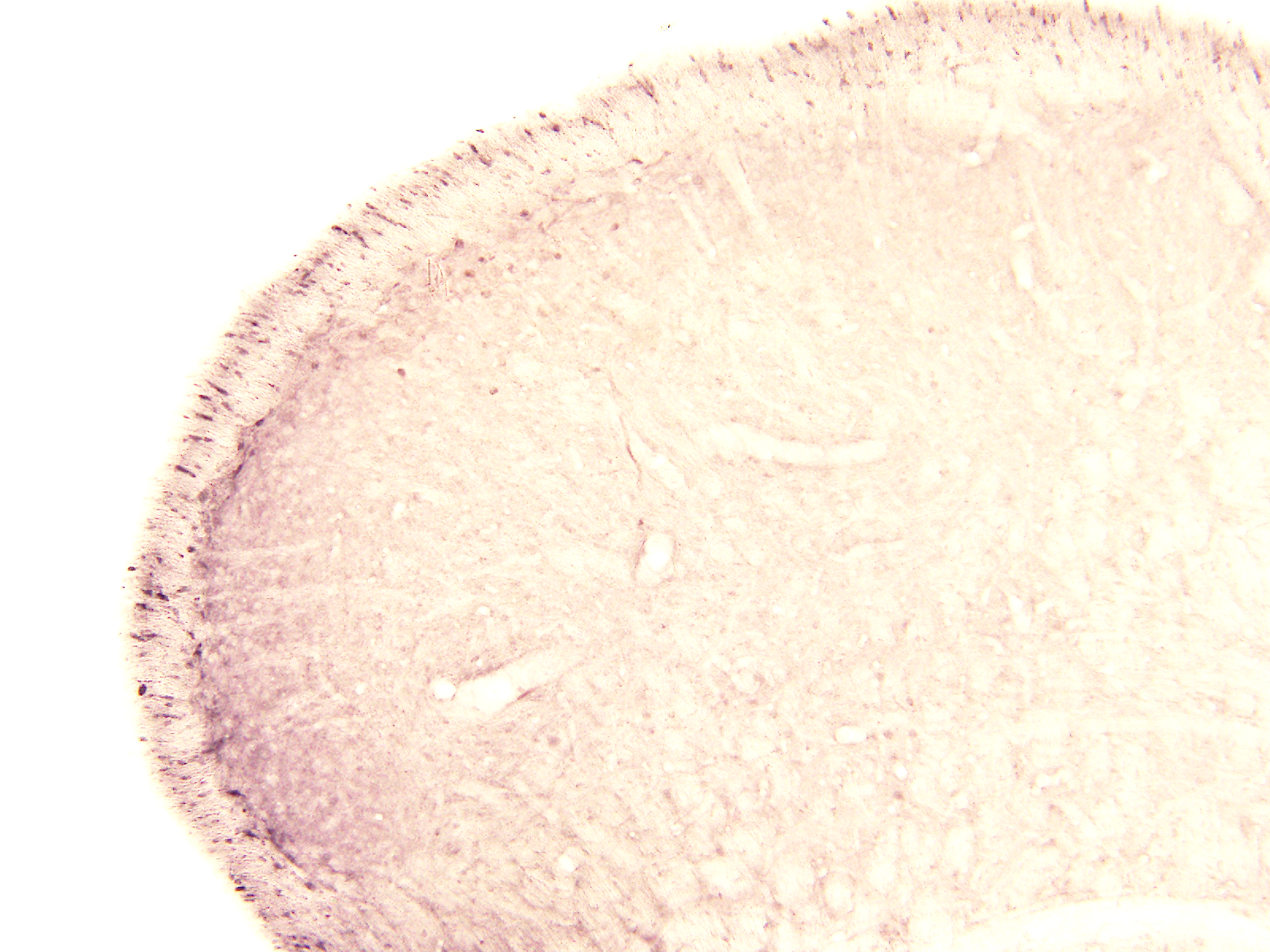

Supplement: Supplementary file 1 [file ijms-21-01945-s001.zip › Supplementary files/Figure S2.TIF]

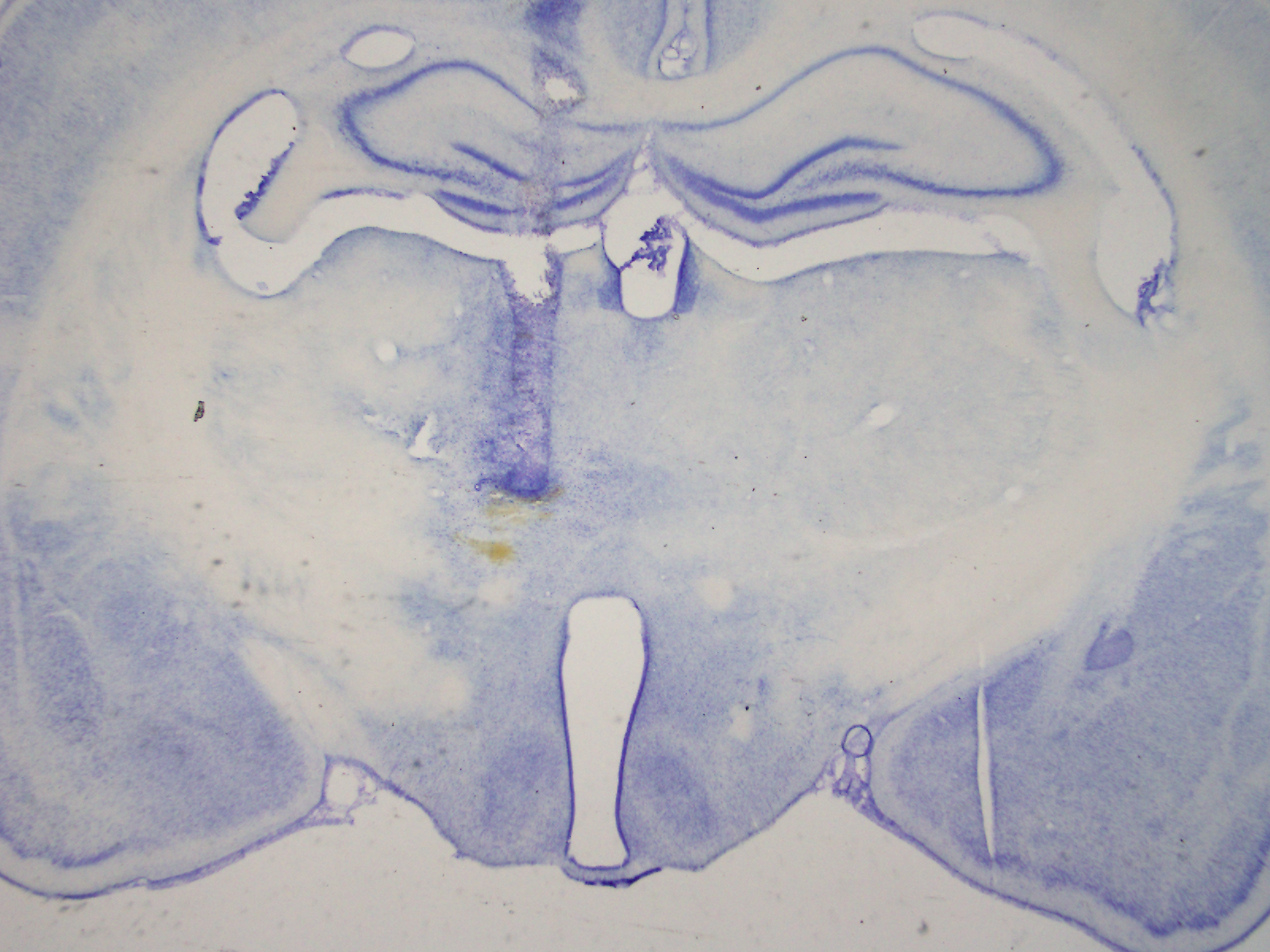

Supplement: Supplementary file 1 [file ijms-21-01945-s001.zip › Supplementary files/Figure S3.TIF]

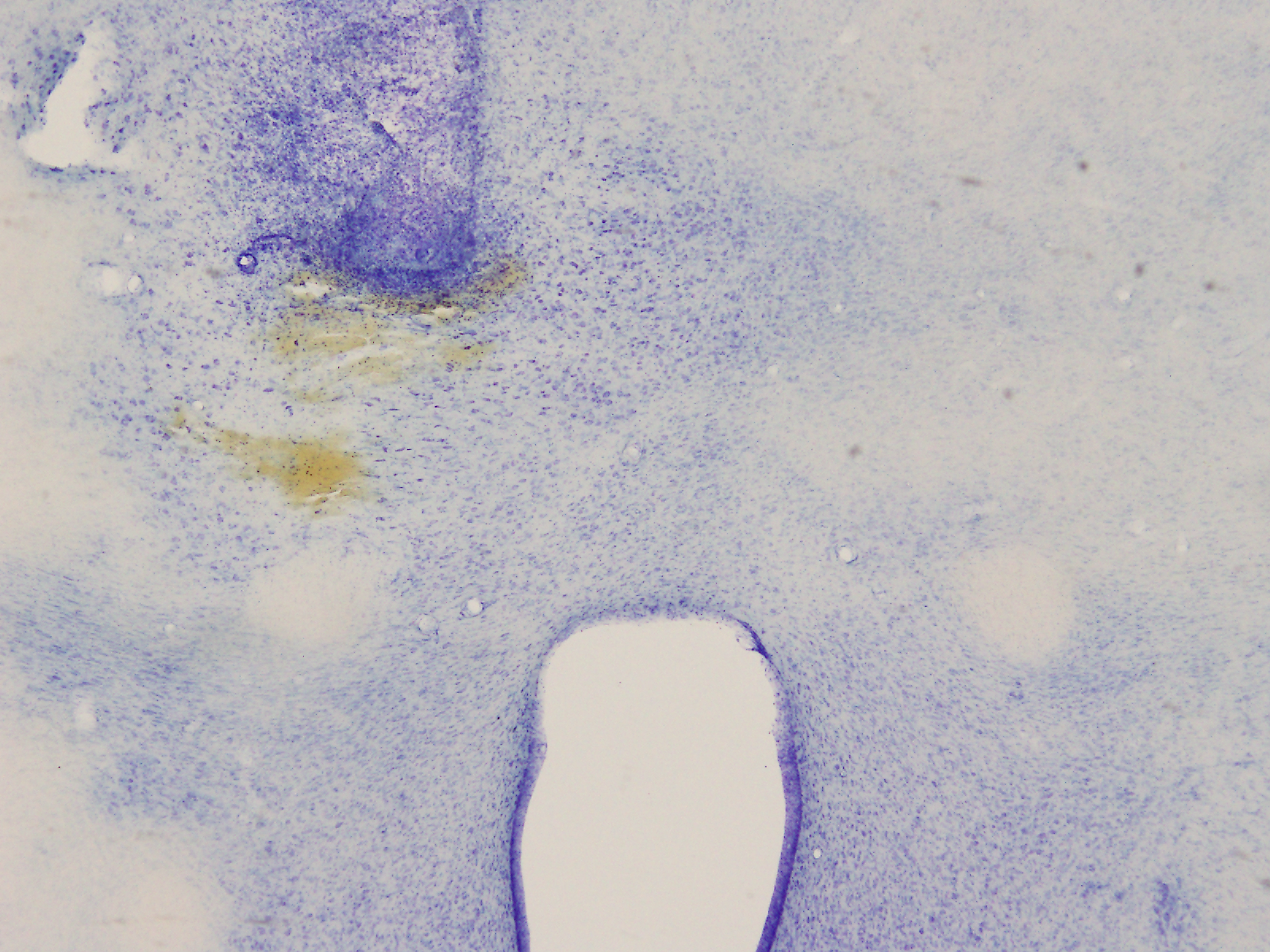

Supplement: Supplementary file 1 [file ijms-21-01945-s001.zip › Supplementary files/Figure S4.TIF]

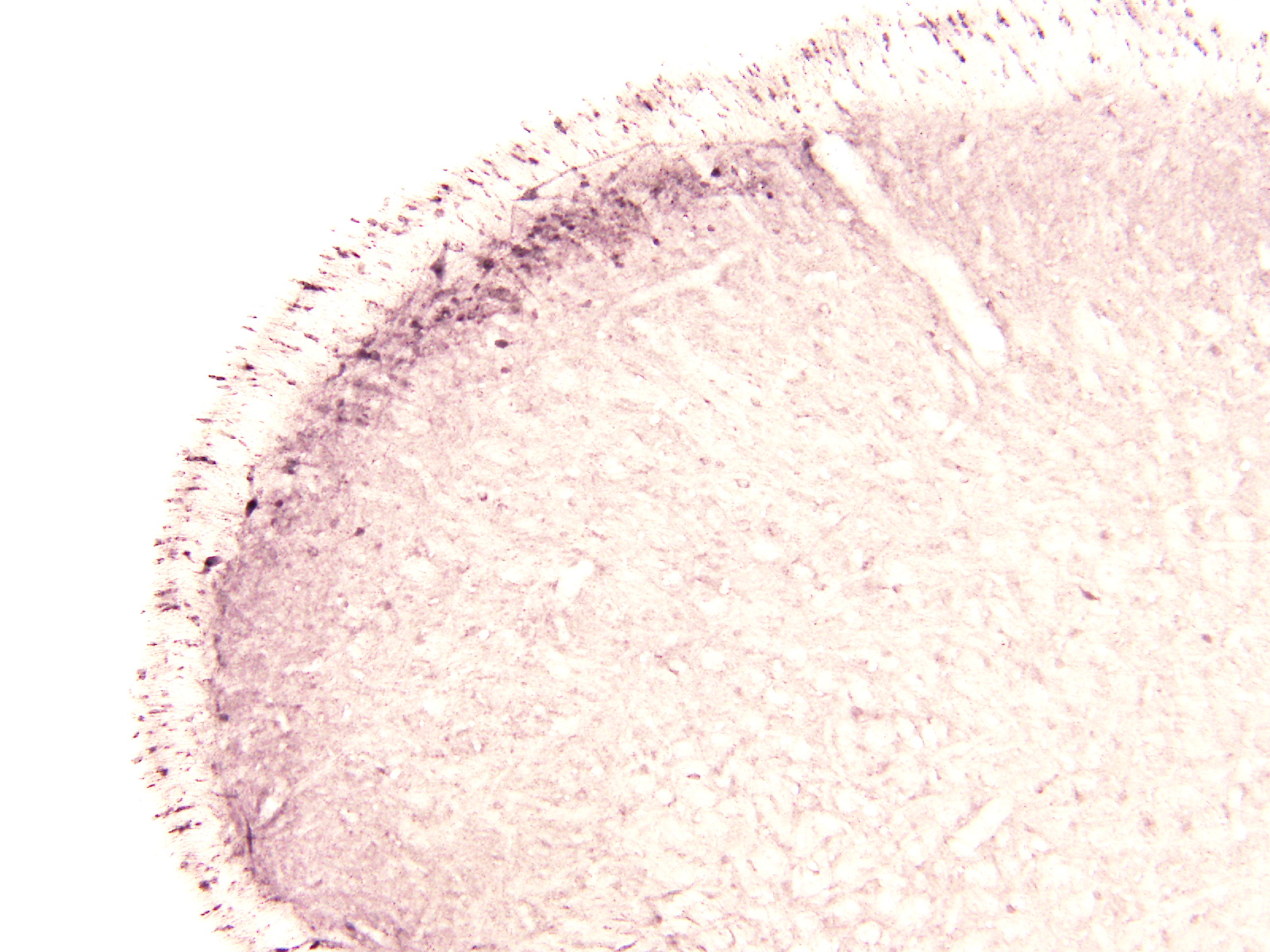

Supplement: Supplementary file 1 [file ijms-21-01945-s001.zip › Supplementary files/Figure S5.TIF]

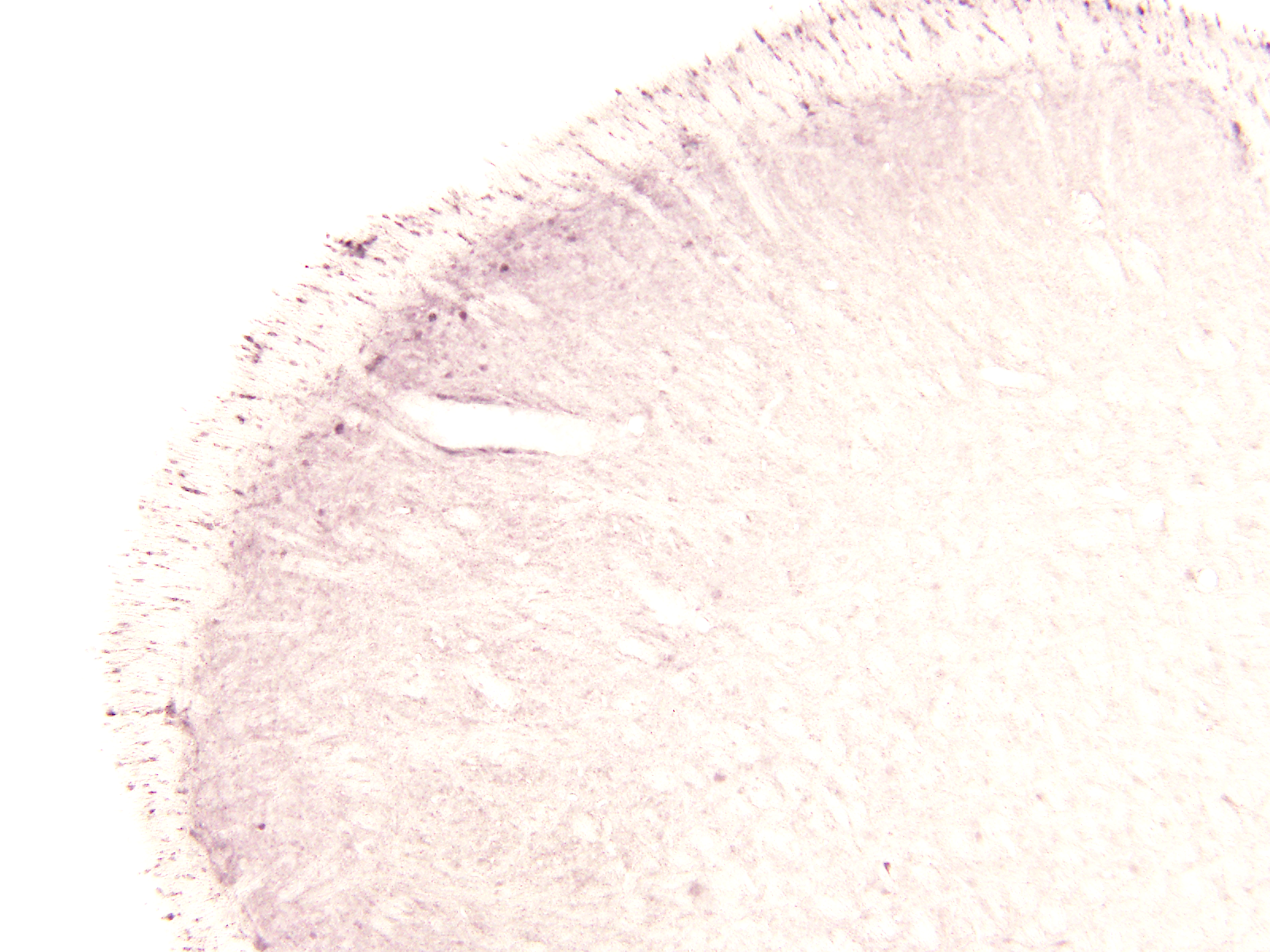

Supplement: Supplementary file 1 [file ijms-21-01945-s001.zip › Supplementary files/Figure S6.TIF]

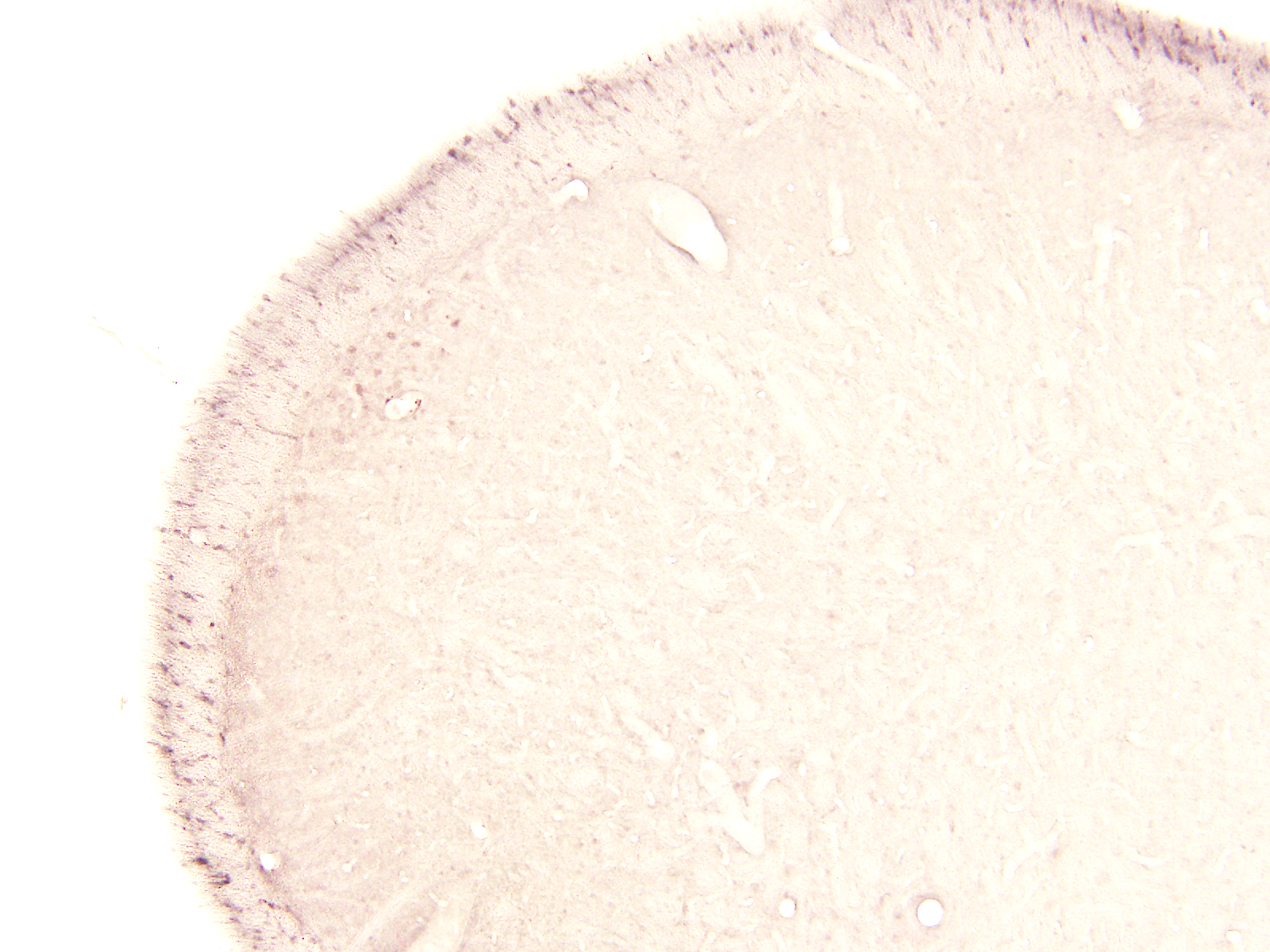

Supplement: Supplementary file 1 [file ijms-21-01945-s001.zip › Supplementary files/Figure S7.TIF]

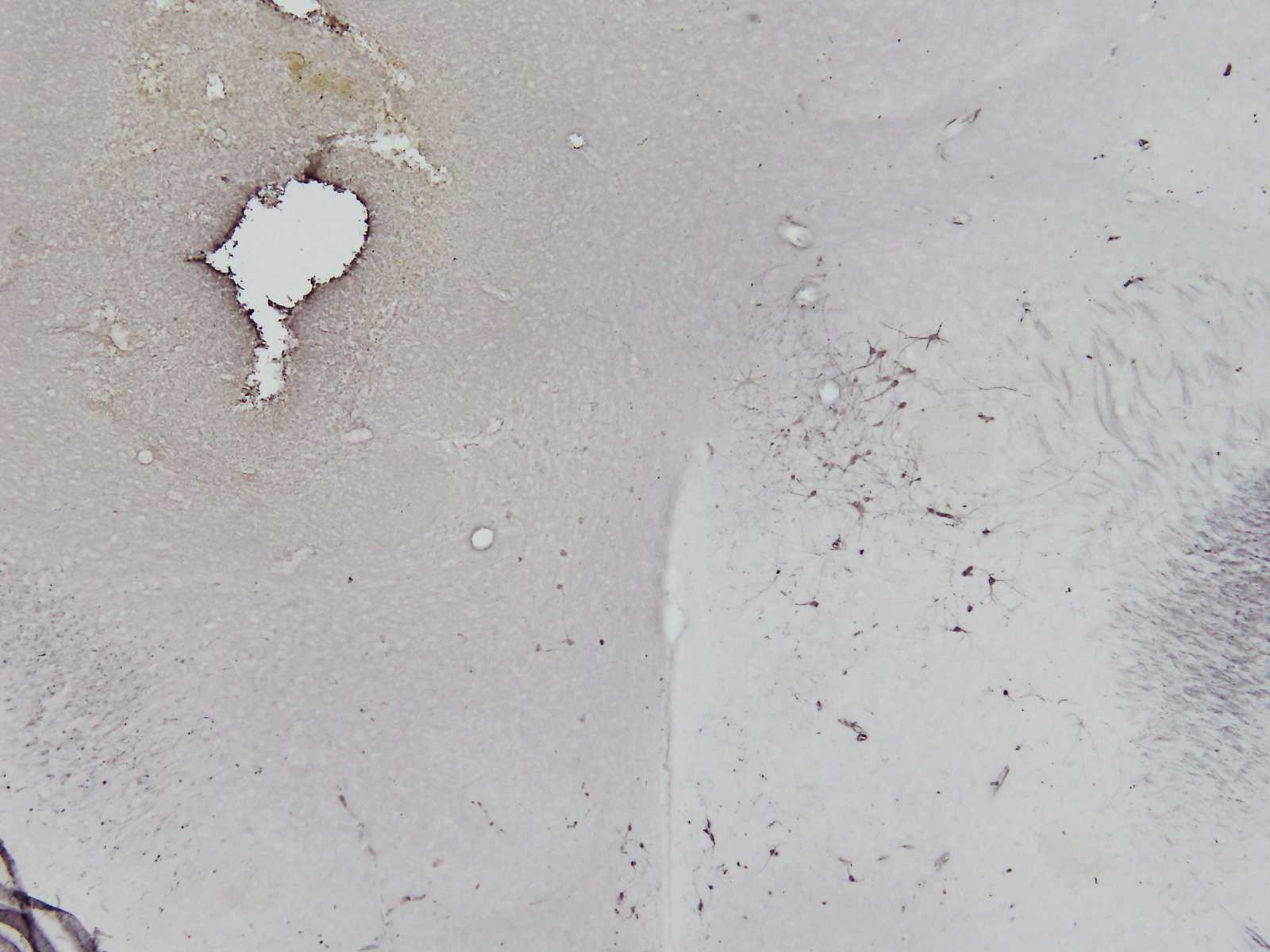

Supplement: Supplementary file 1 [file ijms-21-01945-s001.zip › Supplementary files/Figure S8.TIF]

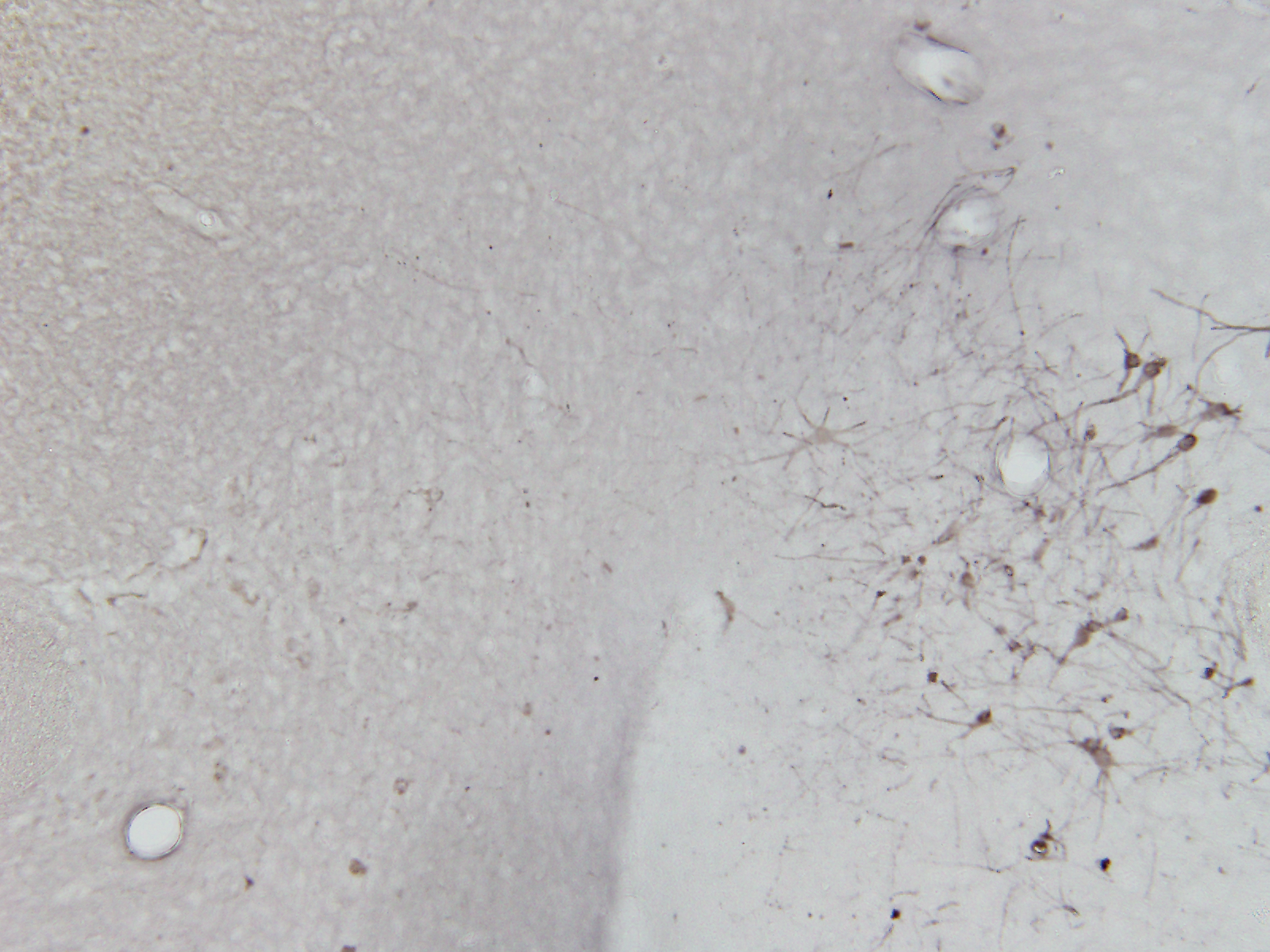

Supplement: Supplementary file 1 [file ijms-21-01945-s001.zip › Supplementary files/Figure S9.TIF]
